# In Vitro Evaluation of the Effect of a Not Cross-Linked Hyaluronic Acid Hydrogel on Human Keratinocytes for Mesotherapy

**DOI:** 10.3390/gels7010015

**Published:** 2021-02-04

**Authors:** Nicola Zerbinati, Sabrina Sommatis, Cristina Maccario, Maria Chiara Capillo, Serena Di Francesco, Raffaele Rauso, Marina Protasoni, Edoardo D’Este, Daniela Dalla Gasperina, Roberto Mocchi

**Affiliations:** 1 Department of Medicine and Surgery, University of Insubria, 21100 Varese, Italy; nicola.zerbinati@uninsubria.it (N.Z.); marina.protasoni@uninsubria.it (M.P.); d.dallagasperina@uninsubria.it (D.D.G.); 2 UB-CARE S.r.l. Spin-off University of Pavia, 27010 Pavia, Italy; sabrina.sommatis@ub-careitaly.it (S.S.); cristina.maccario@ub-careitaly.it (C.M.); mariachiara.capillo@ub-careitaly.it (M.C.C.); serena.difrancesco@ub-careitaly.it (S.D.F.); 3 Maxillofacial Surgery Unit, University of Campania “Luigi Vanvitelli”, 81100 Naples, Italy; dr.raffaele.rauso@gmail.com; 4 Dermatology Department Centro Medico Polispecialistico, 27100 Pavia, Italy; edeste@centro-medico.it

**Keywords:** aging, biorevitalization, Hyaluronic acid (HA), in vitro, mesotherapy, skin

## Abstract

The multicomponent preparations for mesotherapy are based on the principle that skin and hair aging can be prevented by supplying the fundamental substrates for correct cellular functioning, such as nucleotides, vitamins, amino acids, and biomolecules including hyaluronic acid (HA) that promote skin hydration and several biological activities. The study provides evidence for the application of HYDRO DELUXE BIO (Matex Lab S.p.A), a biocompatible hydrogel containing not cross-linked HA, for the treatment of the scalp’s skin by mesotherapy. Using an in vitro model of immortalized human keratinocytes, we studied markers involved in hair aging prevention and growth, such as inflammatory markers, angiogenesis, and oxidative damage. HYDRO DELUXE BIO showed high biocompatibility and the ability to significantly reduce the expression of the inflammation marker interleukin (IL)-8 in Tumor Necrosis Factor (TNF)-stimulated cells. Then, we evaluated angiogenesis, a pivotal event during hair growth, measuring the Vascular Endothelial Growth Factor (VEGF) expression that resulted to be significantly increased in treated cells, suggesting a pro-angiogenetic capability. A protective activity against the oxidative stress agent was showed, increasing the survival rate in treated cells. Concluding, HYDRO DELUXE BIO is suitable for treatment by mesotherapy of the scalp’s skin as it modulates the expression levels of markers involved in the biorevitalization of the hair follicle.

## 1. Introduction

The incidence of hair loss disorders related to healthy scalp alterations is constantly increasing in men and women in relation to the increase in external risk factors. Today, investigation of the molecular processes that underline the alterations of the hair follicles’ homeostasis is the object of interest in the scientific research world, as well as the identification of new molecular targets and possible candidates able to prevent aging of the scalp [1].

The study of hair aging focuses on two main areas: on the one hand, the aesthetic problem of aging hair and loss and its management; on the other hand, the biological events that cause hair loss as microscopic, biochemical, and molecular changes [2]. Most frequently, hair loss is due to a failure to activate existing hair stem cells during hair cycling and could be associated with age. In some forms of alopecia, hair loss is due to genetic mutations in the molecules involved in hair keratin architecture or failure to differentiate properly; they are the most difficult forms to correct. In androgenetic alopecia, hair loss is heritable and androgen-dependent: the genetically predisposed hair follicles are the target for hair follicle miniaturization, leading to a replacement of pigmented hairs by barely visible, depigmented hairs in affected areas up to a decline in visible scalp hair density [3]. Mesotherapy/biorevitalization is a minimally invasive technique that consists of local intradermal therapy (LIT) with medical devices or other substances [4]. This approach is commonly used as a treatment for the rejuvenation of the skin but also of the hair follicle subjected to intrinsic, but also extrinsic aging. Smoke, environmental pollution, ultraviolet radiation, and lifestyle are the most common stress factors associated to extrinsic aging [5]. Aging related hair loss is also associated with individual genetic predisposition and epigenetic mechanisms. It could be hereditable, like androgen-dependent alopecia (AGA), and related to dihydrotestosterone (DHT) biological pattern [2,6].

The development of complex multicomponent preparations for mesotherapy is based on the principle that skin and hair aging can be prevented by supplying the various substrates that are fundamental for correct cellular functioning. Therefore, the main constituents of these preparations are usually nucleotides, vitamins, minerals, amino acids, coenzymes, antioxidants, and biomolecules such as hyaluronic acid (HA), an anionic non-sulfated glycosaminoglycan widely distributed in connective, epithelial, and neural tissues [7].

The introduction of HA-based formulations is a current way to exploit the intrinsic features of this biomolecule such as to promote skin hydration, as well as anti-inflammatory, antibacterial, antimycotic, and antioxidant activity. These properties provide an optimal environment for biochemical processes and energetic metabolism, protecting from extrinsic aging-related risk factors [8,9]. Experimental evidence supports the hypothesis that oxidative stress and the resulting state of inflammation play a major role in premature skin and hair aging [2].

Hair follows a specific growth cycle with anagen (growing phase), catagen (involution phase or transition phase) and telogen (resting phase) phases [10]; the scalp follicle is a multicellular complex composed of Follicle Dermal Papilla Cells (FDPCs), inner and outer root sheath cells (IRS and ORS), melanocytes, and keratinocytes. FDPCs are related to hair proliferation with normal and onset conditions; instead, keratinocytes play a crucial role in hair growth, producing hair fibers composed of 10 nm keratin filaments [11]. The region of the hair shaft in which keratinocytes rapidly proliferate is called the “hair matrix area” and surrounds the dermal papilla; the correct interaction between keratinocytes and dermal papilla cells in this region provides essential signals for both follicle induction and hair growth [2].

In this study, we present in vitro results regarding the effect of the product HYDRO DELUXE BIO (Matex Lab SpA, Via Carlo Urbani 2, ang Enrico Fermi, Brindisi, Italy), a highly biocompatible [12,13,14] hydrogel containing not cross-linked hyaluronic acid for mesotherapeutic use. The innovative formulation presents a wide range of amino acids (Lysine, Proline, Glycine, Leucine, Isoleucine, Valine, Serine, and Alanine) and biomolecules such as Carnosine and reduced Glutathione (GSH), whose main property is to stimulate the anabolic functions of scalp cells such as replication, protein synthesis, production of extracellular matrix components (ECM), and promoting the growth of hair follicles. The experiments were carried out in order to determine the in vitro effect of the product on an immortalized human keratinocytes model, evaluating cell viability and the effect on mechanisms involved in hair growth and in hair aging prevention, such as inflammatory markers, angiogenesis, and oxidative damage.

## 2. Results and Discussion

### 2.1. Evaluation of the Cytotoxicity by MTT Assay

A cytotoxicity test is required to evaluate the effect of HYDRO DELUXE BIO on cell viability and to determine the appropriate concentrations required for the following assays. The product HYDRO DELUXE BIO does not show cytotoxic activity after treatment with all the tested concentrations on human keratinocytes after 24 h of treatment (80–0.625 mg/mL). The same test was performed by dissolving the product in medium with different Fetal Bovine Serum (FBS) concentrations (10% and 0.5%) and in all conditions, the results were confirmed (viability ≥ 70% at all concentrations, data not shown).

### 2.2. Modulation of Inflammatory Markers

**Figure 1:**
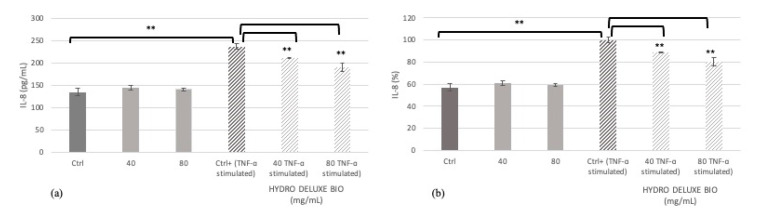
HYDRO DELUXE BIO treatment induces a decrease in IL-8 levels after TNF-α stimulation. The expression levels of IL-8 were determined in HaCat cells by ELISA assay. Values are expressed as concentration in pg/mL (**a**) and as percentage (**b**) on HaCaT cells. Ctrl: untreated cells; 40: cells treated with HYDRO DELUXE BIO 40 mg/mL; 80: cells treated with HYDRO DELUXE BIO 80 mg/mL; Ctrl+: untreated cells + TNF-α; HYDRO DELUXE BIO: cells treated with 40 or 80 mg/mL + TNF-α. Values of ** *p* ≤ 0.01 were considered highly significant compared with Ctrl+ by one-way ANOVA statistical analysis.

The possible activity on inflammatory cytokines of the tested product HYDRO DELUXE BIO after stimulation with Tumor Necrosis Factor (TNF)-α was evaluated in HaCaT cells, measuring the expression of interleukin (IL)-8 by an ELISA technique. The IL-8 levels were expressed as concentration in pg/mL and percentage in HaCaT cells treated with the tested medical device at the concentrations of 40 and 80 mg/mL compared to positive control (Ctrl+, cells treated only with TNF-α) (Figure 1 and Table 1).

The values are reported as average ± SD of the concentrations derived from samples’ absorbance values, interpolating them with the standard curve according to the manufacturer’s instructions.

The reported data highlight that treatment with the product HYDRO DELUXE BIO significantly reduces the release of IL-8 in normal human keratinocytes in inflammatory conditions. In particular, the inhibition percentage of interleukin release is equal to 11.01% and 20.03% after treatment with 40 and 80 mg/mL of the product, respectively, indicating a highly significant modulation of the inflammatory cytokine exerted by the tested product. Treatment with HYDRO DELUXE BIO in the absence of TNF-α stimulation provided IL-8 levels comparable to control cells.

### 2.3. Evaluation of Vascular Endothelial Growth Factor (VEGF) Release

VEGF plays an important role in the angiogenesis process during the hair growth cycle [15]. Stimulation of VEGF by HaCaT cells supports hair growth, promoting the potential of the tested product.

**Figure 2:**
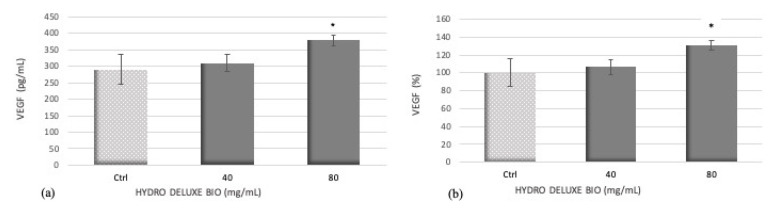
HYDRO DELUXE BIO treatment induces an increase in VEGF secretion at the highest tested concentration. Effect of 24 h treatment with the product HYDRO DELUXE BIO on VEGF secretion expressed as concentration in pg/mL (**a**) and as percentage (**b**). Ctrl: untreated cells; HYDRO DELUXE BIO: treated cells with 40 or 80 mg/mL. Values of * *p* ≤ 0.05 were considered statistically significant compared with Ctrl, following the statistical analysis with the one-way ANOVA method.

VEGF secretion is expressed as concentration in pg/mL and percentage in HaCaT cells after 24 h of treatment with the product HYDRO DELUXE BIO at the concentrations of 40 and 80 mg/mL compared to untreated cells (Ctrl) (Figure 2 and Table 2).

The values are reported as average ± SD of the samples’ absorbance values, interpolating them with the standard curve according to the manufacturer’s instructions.

Treatment of HaCaT cells with the tested product HYDRO DELUXE BIO at 40 and 80 mg/mL, resulted in a dose-dependent increase in VEGF secretion. In particular, only the treatment with the highest tested concentration (80 mg/mL) induces a statistically significant increase of about 30% compared with untreated cells (* *p* ≤ 0.05).

### 2.4. Protection against Oxidative Damage

**Figure 3:**
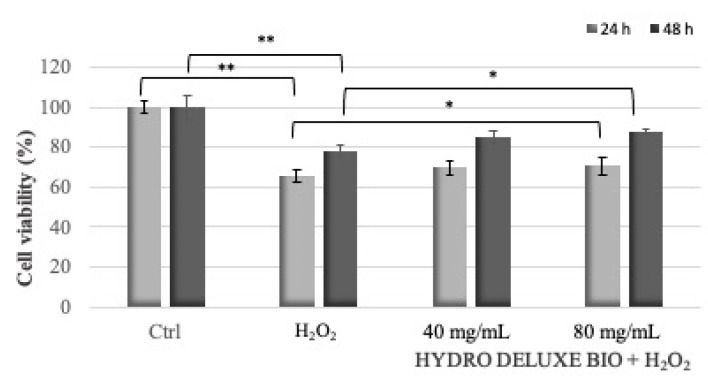
Protective effect of the product HYDRO DELUXE BIO against H_2_O_2_-induced oxidative damage. HaCaT cells were co-treated with 40 and 80 mg/mL and H_2_O_2_ used as oxidative stress. 24 h: treatment with the tested product + H_2_O_2_ 0.125 mM; 48 h: treatment with the tested product + H_2_O_2_ 0.0625 mM. * *p* ≤ 0.05 ** *p* ≤ 0.01 were considered, respectively, to be statistically significant when compared with H_2_O_2_ by one-way ANOVA statistical analysis.

The protective effect of HYDRO DELUXE BIO against H_2_O_2_-induced cell death in HaCaT cells was estimated by cells’ viability through an MTT test. HaCaT cells were treated with 0.125 and 0.0625 mM H_2_O_2_ (for 24 and 48 h treatment, respectively) together with the tested product at the concentrations of 40 and 80 mg/mL to evaluate the protection against oxidative damage (Figure 3 and Table 3).

The product HYDRO DELUXE BIO showed a protective effect against H_2_O_2_-induced cell oxidative damage. The results suggest that the damage induced by H_2_O_2_ in HaCaT cells is decreased in a significant way by the product only for the highest concentrations tested.

HYDRO DELUXE BIO is a product for mesotherapeutic use (Matex Lab SpA, Via Carlo Urbani 2, ang Enrico Fermi, Brindisi, Italy), containing components considered to enhance hair follicle biorevitalization and to provide anti-aging activity. This study was designed to evaluate the biocompatibility and the effect on inflammatory markers, on angiogenesis, and on the protection from oxidative stress of the tested product. Hair loss, alopecia, dandruff, and other scalp disorders are often the result of an alteration in the normal hair cycle, dependent on a complex network of grow factors, hormones, and other signaling markers involved in proliferation and apoptosis [15].

The first aim of this study was to better characterize a defined set of skin and hair surface biomarkers after treatment with the product. Firstly, the biocompatibility of the product was evaluated with the primary purpose to determinate the appropriate concentrations to use in the efficacy assays. The results highlighted the high biocompatibility of the product, which resulted to be not cytotoxic at all the tested concentrations. Then, the efficacy tests were performed at the highest tested concentrations with the best solubility (40 and 80 mg/mL).

Hair loss, alopecia, and dandruff lesions are often associated with epidermal hyperplasia and mild inflammation. Inflammation biomarkers reflect a biochemical alteration on the scalp surface and their quantification is the index of a healthy stratum corneum (SC) [16]. In particular, we examined IL-8 levels, a hallmark indicator of a wide variety of inflammation responses and a phenomenon that is significantly present in scalp alterations with respect to baseline conditions. Our results showed that the product HYDRO DELUXE BIO is able to significantly reduce IL-8 levels, demonstrating an effective modulation of inflammation. In particular, the highly significant reduction is equal to 11.01% and 20.03% after treatment with 40 and 80 mg/mL of the tested product, respectively.

Angiogenesis is another process that was investigated because it represents a pivotal event during hair growth cycle, and in particular, follicle-derived VEGF is responsible for an increase in blood supply to the tissues stimulating proliferation and hair growth-related differentiation [15]. VEGF expression in HaCat cells is investigated in the presence of HYDRO DELUXE BIO as a marker of angiogenesis. We observed that treatment of HaCaT cells with the product at the highest tested concentrations of 80 mg/mL resulted in a remarkable increase in VEGF levels. In particular, the treatment with the product at the concentration of 80 mg/mL determines a significant increase in VEGF release equal to 34%, demonstrating a stimulation of an angiogenetic-related marker.

Moreover, the increased levels of reactive oxygen species (ROS) in scalp tissue are one of the primary causes of scalp disorders [17]. This study demonstrated the protective effect of the product HYDRO DELUXE BIO against H_2_O_2_-induced cellular damage in HaCaT cells was. In particular, the performed analyses demonstrated that a pre-treatment of the cells was able to significantly increase the survival rate significantly for the cells treated with the product’s concentration of 80 mg/mL.

In the literature, studies regarding the effects of mesotherapy and biorevitalization products are reported. The aim of these products is to maintain or restore the healthy texture of the skin/hair. Firm, bright, and moisturized skin is obtained by application on the superficial dermis of suitable products that are perfectly biocompatible and totally absorbable [18].

Several experimental studies demonstrated that hyaluronic acid (HA) injected into the skin can stimulate fibroblasts to express collagen type 1 (Col-1), matrix metalloprotease-1 (MMP-1), and tissue inhibitor of matrix metalloproteinase-1 (TIMP) [19], thus promoting hydration and fibroblasts activation [20,21]. Collagen production in skin cells is also stimulated by injection of vitamins [22] and antioxidant substances are able to reverse aging, as one of the most studied hypotheses regarding aging is that it is caused by oxidative stress, because oxidation can damage proteins, DNA, and lipids.

Recent clinical and in vitro studies demonstrated that a formulation rich in HA, vitamins, and amino acids interfered with the effects on collagen production in damaged skin by increasing the expression of Col-1. Then, downregulation of IL-1β, IL-6, and MMP1 suggested a role in the inflammatory processes [23,24].

The positive role of HA formulations was also demonstrated in other cell lines such as human skin fibroblasts, with an increase in the expression of type I collagen and elastin and a decrease in MMP-1, IL-6, and IL-1 levels [4].

In conclusion, the present study identifies the product HYDRO DELUXE BIO as a useful candidate for biorevitalization of the hair follicle and the scalp’s skin.

## 3. Materials and Methods

### 3.1. Cell Culture and Sample Preparation

Keratinocytes are the most represented cell type in the epidermis. They grow from the base of the epidermis where cells multiply, and then migrate to the surface of the skin producing lipids, natural factors of hydration, and keratin. The cell culture used in this study is a human immortalized keratinocytes cell line (HaCaT, code BS CL 168). The cell culture was grown in conditions of complete sterility and maintained in incubation at 37 °C with 5% carbon dioxide (CO2) atmosphere.

The product HYDRO DELUXE BIO was weighed and dissolved at the concentration of 80 mg/mL in complete medium constituted by Dulbecco’s modified Eagle’s medium (DMEM; Biowest) supplemented with fetal bovine serum (FBS; Gibco-Fisher Scientific, Waltham, MA, USA), 1 mM L-glutamine (Capricorn Scientific, Ebsdorfergrund, Germany), and antibiotics (100 U/mL penicillin and 100 μg/mL streptomycin; Capricorn Scientific, Ebsdorfergrund, Germany).

### 3.2. Cytotoxicity Assay (MTT Test)

For the preparation of the assay, HaCaT cells were homogeneously seeded into 96-well plates at a density of 2 × 10^4^ cells per well and incubated at 37 °C with 5% CO_2_ humidified atmosphere. After 24 h, cells were treated with the tested medical device (3 replicates for each dilution), starting from 80 mg/mL, prepared directly in culture medium and following serial dilution (1:2). Untreated cells were used as control. After 24 h of treatment, the culture medium was removed and cells were incubated with 50 µL of MTT solution (1 mg/mL) at 37 °C for 2 h. Subsequently the supernatant was removed and 100 µL of isopropanol was added to dissolve formazan products. Absorbance was determined at 570 nm wavelength using a microplate reader. Cell survival was calculated measuring the difference in optical density of the tested product with respect to control (untreated cells) [25].

Cell viability (%) = [OD570 nm test product/OD570 nm negative control] × 100

### 3.3. Modulation of Inflammatory Markers

Enzyme-linked immunosorbent assay (ELISA) has been the method of choice for the quantification of cytokines and chemokines release in cell culture supernatants samples [26].

The content of interleukin (IL)-8 after treatment with the tested product HYDRO DELUXE BIO was evaluated in HaCaT cells, after 24 h of treatment, using an ELISA assay kit (Thermo Fisher, Waltham, MA, USA) according to the manufacturer’s instructions.

For preparation of the assay, cells were homogeneously seeded into a 96-well plate at a density of 2 × 10^4^ cells per well and incubated at 37 °C with 5% CO_2_ humidified atmosphere. On the following day, cells were washed in PBS and the medium was replaced with the product at the not cytotoxic concentrations determined by the MTT test at 0.5% FBS; cells were stimulated with Tumor Necrosis Factor alpha (TNF-α) during the last 6 h of treatment. A non-stimulated control condition and a stimulated control condition without the product were also performed in parallel. Cells not stimulated and treated with HYDRO DELUXE BIO represent a control of IL-8 basal levels.

At the end of the treatment, supernatants were collected and used for the coating of a specifically pre-treated 96-well ELISA plate provided by the kit. The standards were reconstituted and used to perform the standard curve (1000–15.6 pg/mL). Samples, blanks, and standards were added to each well in duplicate and the assay was performed according to the supplier’s instructions.

The absorbance was then measured at 450 nm using a microplate reader (Multiskan, Thermo Scientific, Waltham, MA, USA). Data were analyzed as mean ± standard deviation (SD) (pg/mL of IL-8/µg protein). The modulatory activity was evaluated in terms of percentage of inhibition obtained by comparison of the mean values of control and treated conditions.

% inhibition = 100 − (mean value of treated condition/mean value of untreated condition) × 100.

### 3.4. Evaluation of Vascular Endothelial Growth Factor (VEGF) Levels

Angiogenesis is another process that represents a pivotal event during hair growth, and in particular, follicle-derived VEGF is responsible of an increase in blood supply to the tissues, stimulating proliferation and hair growth-related differentiation [15].

Pro-angiogenic efficacy was determined by measuring the VEGF levels in extracellular medium by a Quantikine Human ELISA assay kit (R&D Systems, Minneapolis, MN, USA).

To perform the test, cells were plated in a 6-well culture plate and treated with the tested product at the concentrations determined by the MTT test at 1% FBS. At the end of the treatment, supernatants were collected and the secretion of VEGF into extracellular medium was evaluated according to the ELISA kit manufacturer’s instructions. Human VEGF standard was reconstituted and used to build the standard curve (1000–15.6 pg/mL). Standards and samples were added in duplicate into wells and the VEGF amount was measured plotting the log of the human VEGF concentrations versus the log of the O.D.; the best-fit line was obtained by regression analysis [15].

Effect of VEGF release was calculated as:

% increase in VEGF = [(C−D)/D] × 100

where:

C = concentration of VEGF (pg/mL) in cells treated with the tested product;

D = concentration of VEGF (pg/mL) in untreated cells.

### 3.5. Evaluation of the Cell Protection against Oxidative Damage

To evaluate the protective effect against oxidative stress in human keratinocytes after treatment with HYDRO DELUXE BIO, preliminary experiments were performed in order to identify the concentration of hydrogen peroxide (H_2_O_2_) able to cause a decrease in cell respiration (index of cell viability) ranging from 30 to 40% by the MTT test.

For preparation of the assay, cells were homogeneously seeded into a 96-well plate at a density of 1.5 × 10^4^ cells per well and incubated at 37 °C, with 5% CO_2_ humidified atmosphere. HaCaT cells were then co-treated with H_2_O_2_ and the tested medical device at the non-cytotoxic concentrations. Survival of cells against H_2_O_2_-induced cytotoxicity was monitored after 24 and 48 h by MTT assay.

Protective effect was determined as restoration of cell viability [15]:

% cell viability = 100 − (% cytotoxicity)

where:

% cytotoxicity = (A−B/A) × 100;

A = absorbance of untreated cells;

B = absorbance of cells treated with H_2_O_2_ alone or H_2_O_2_ + tested concentrations.

Statistical significance was determined using the One -way Anova with Fisher’s LSD post test and *p*-values of less than 0.05 were considered statistically significant (* *p* ≤ 0.05 and ** *p* ≤ 0.01).

## Figures and Tables

**Table 1:** Values of IL-8 levels, expressed as concentration and percentage (mean ± SD), in cells after treatment with the product HYDRO DELUXE BIO.

Sample	IL-8 (pg/mL)	IL-8 (%)
Ctrl	135.21 ± 7.74	56.92 ± 3.26
HYDRO DELUXE BIO (180515-30) 40 mg/mL	144.30±5.30	60.75 ± 2.23
HYDRO DELUXE BIO (180515-30) 80 mg/mL	141.16 ± 2.84	59.42± 1.19
Ctrl + (TNF-α stimulated)	237.55 ± 5.57	100.00± 2.35
HYDRO DELUXE BIO (180515-30) (TNF-α stimulated) 40 mg/mL	211.36 ± 0.69	88.98 ± 0.29
HYDRO DELUXE BIO (180515-30) (TNF-α stimulated) 80 mg/mL	189.96 ± 9.17	79.97 ± 3.86

**Table 2:** Values of VEGF levels, expressed as concentration and percentage, in HaCaT cells after treatment with the product HYDRO DELUXE BIO compared to control.

Sample	VEGF (pg/mL)	VEGF (%)
Ctrl	285.05 ± 33.47	100 ± 11.74
HYDRO DELUXE BIO (180515-30) 40 mg/mL	309.86 ± 17.82	108.71 ± 6.25
HYDRO DELUXE BIO (180515-30) 80 mg/mL	391.90 ± 26.43	137.49± 9.27

**Table 3:** Percentage changes (mean ± SD) in cell viability after 24 and 48 h of treatment with the oxidative stressor and the product HYDRO DELUXE BIO at the two non-cytotoxic concentrations of 40 and 80 mg/mL. *H_2_O_2_ was used at two different concentrations, 0.125 mM for 24 h treatment and 0.0625 mM for 48 h treatment.

Sample	24 h	48 h
H_2_O_2_*	65.40 ± 3.26	78.18 ± 3.28
H_2_O_2_* + HYDRO DELUXE BIO (180515-30) 40 mg/mL	69.95 ± 3.49	84.83 ± 3.02
H_2_O_2_* + HYDRO DELUXE BIO (180515-30) 80 mg/mL	70.62 ± 4.16	87.57 ± 1.53

## Data Availability

Data are included in the text; raw data are available from the corresponding author.
